# Supplementary material for: ROCK signaling promotes collagen remodeling to facilitate invasive pancreatic ductal adenocarcinoma tumor cell growth
Source: EMBO Mol Med. 2016 Dec 28;9(2):198–218. doi: 10.15252/emmm.201606743 (PMC5286371; doi:10.15252/emmm.201606743)
Supplement: Supplementary file 1 — Appendix [file EMMM-9-198-s001.pdf]

Appendix - ROCK signaling promotes collagen remodeling to facilitate invasive pancreatic ductal adenocarcinoma tumor cell growth.

Nicola Rath, Jennifer P Morton, Linda Julian, Lena Helbig, Shereen Kadir, Ewan J McGhee, Kurt I Anderson, Gabriela Kalna, Margaret Mullin, Andreia V Pinho, Ilse Rooman, Michael S Samuel, Michael F Olson

Table of contents

Appendix Supplementary Methods

- Primary pancreatic exocrine cultures
- Attachment assay

Appendix Supplementary Figures

- Appendix Figure S1 - ROCK2 expression in mouse pancreata
- Appendix Figure S2 - Tissue selective expression of conditionally-regulated ROCK2
- Appendix Figure S3 - Conditional ROCK activation in KC mice does not alter PanIN numbers
- Appendix Figure S4 - Invasion of KPC or KPflC cells into fibroblast conditioned collagen
- Appendix Figure S5 - ROCK-induced substrate phosphorylation and changes in cell morphology
- Appendix Figure S6 - Average RNA sequence reads for indicated collagens with >10 copies detected
- Appendix Figure S7 - Analysis of KPC mouse tumors with Fasudil ROCK inhibitor treatment

Tables Supplementary Tables

- Supplementary Table S1 - ROCK-induced changes in gene expression associated with Metacore^{TM} gene process networks
- Supplementary Table S2 - RNA sequencing reads for Mmp and Timp transcripts
- Supplementary Table S3 - RNA sequencing reads for collagen transcripts
- Supplementary Table S4 - Clinical information data provided by US Biomax for Pancreas cancer tissue array (PA961c)

References

Appendix Supplementary Methods

Primary pancreatic exocrine cultures

Pancreatic exocrine cells were isolated as previously described(Pinho *et al*, 2011). Pancreata were injected with 2.5 ml of a 1.25 mg/ml Collagenase P (Roche)/HBSS (Gibco) solution and after removal from the mouse, placed into 5 ml of Collagenase P/HBSS solution. The tissue was cut into small pieces and digested in a shaking water bath at 37°C for 20 min. The reaction was stopped by placing the solution on ice. 10 ml cold HBSS + 5% FBS was added and after sedimentation of the cellular fraction, the supernatant was aspirated. Cell pellets were washed three times with 10 ml HBSS + 5% FBS. The cell suspension was centrifuged twice for 2 min at 1000 rpm and then the cell suspension was filtered sequentially through a 500 μm polypropylene mesh (Spectrum laboratories) and a 100 μm nylon filter (BD Falcon). Next, the cell suspension was layered on top of a 20 ml HBSS + 30% FBS solution and viable acinar cells pelleted by centrifugation for 2 min at 1000 rpm. Cells were cultured in defined media: DMEM/F12 supplemented with 3% FBS (Sigma), 1 mM Pyruvate (Gibco), 1x Non-essential amino acids (Gibco), 1x N2 supplement (Gibco), 0.5x B27 supplement (Gibco), 0.1% β-mercaptoethanol (Gibco), 20 ng/ml EGF (R&D systems), 10^3 U/mL Esgro mLIF (Millipore), 25 μg/ml G418 (10131-027, Gibco), 0.1 mg/ml Soybean Trypsin Inhibitor (T6522, Sigma), and antibiotics (Penicillin-Streptomycin, 15070-063, Gibco)(Pinho *et al*, 2011). As indicated, EtOH vehicle, 2 μM 4HT or 10 μM Y27632 were added to the medium.

Attachment assay

Primary exocrine cells were seeded into 24-well plates and allowed to attach over a period of 48 h. Attachment of primary acinar cells was measured using the

Sulforhodamine B (SRB) assay. Cells were fixed to tissue culture plates with 1% trichloroacetic acid, stained with 0.4% SRB/1% glacial acetic acid for 45 min, and then washed 4 times with 1% acetic acid. Plates were left to dry overnight. Next day, 500 μl of 10 mM Tris Base was added to each well and plates were left on a shaker for 15 min. For *Pdx1-Cre; LSL-KRas*$^{G12D/+}$ primary cultures 75 μl and for *Pdx1-Cre* and *Pdx1-Cre; LSL-ROCK2:ER* primary cultures 100 μl were transferred to a 96-well-plate and absorbance was measured at 570 nm in triplicate.

Appendix Figure S1 - ROCK2 expression in mouse pancreata (related to Fig 1).

A Validation of ROCK2 antibody specificity using serial sections of pancreatic tissue from *Pdx1-Cre; Rosa26-LSL-RFP; ROCK2*$^{fl/fl}$ mice. Serial tissue sections were immunohistochemically stained for RFP or ROCK2. Images reveal mosaic activity of the Pdx1-Cre recombinase as indicated by RFP expression. The ROCK2 antibody stained RFP negative cells that had not recombined the *ROCK2*$^{fl/fl}$ locus. Scale bar = 100 µm.

B *Pdx1-Cre; Rosa26-LSL-RFP; ROCK2*$^{fl/fl}$ cohorts were aged for 25 weeks. No significant effect of Pdx1-Cre mediated ROCK2 deletion was observed by comparing ROCK2$^{+/+}$, ROCK2$^{+/fl}$ and ROCK2$^{fl/fl}$ mice for body weight or pancreas/body weight, for males or females. Scatter plots show means ± SD (n=6-15), p-values determined by Kruskal-Wallis test.

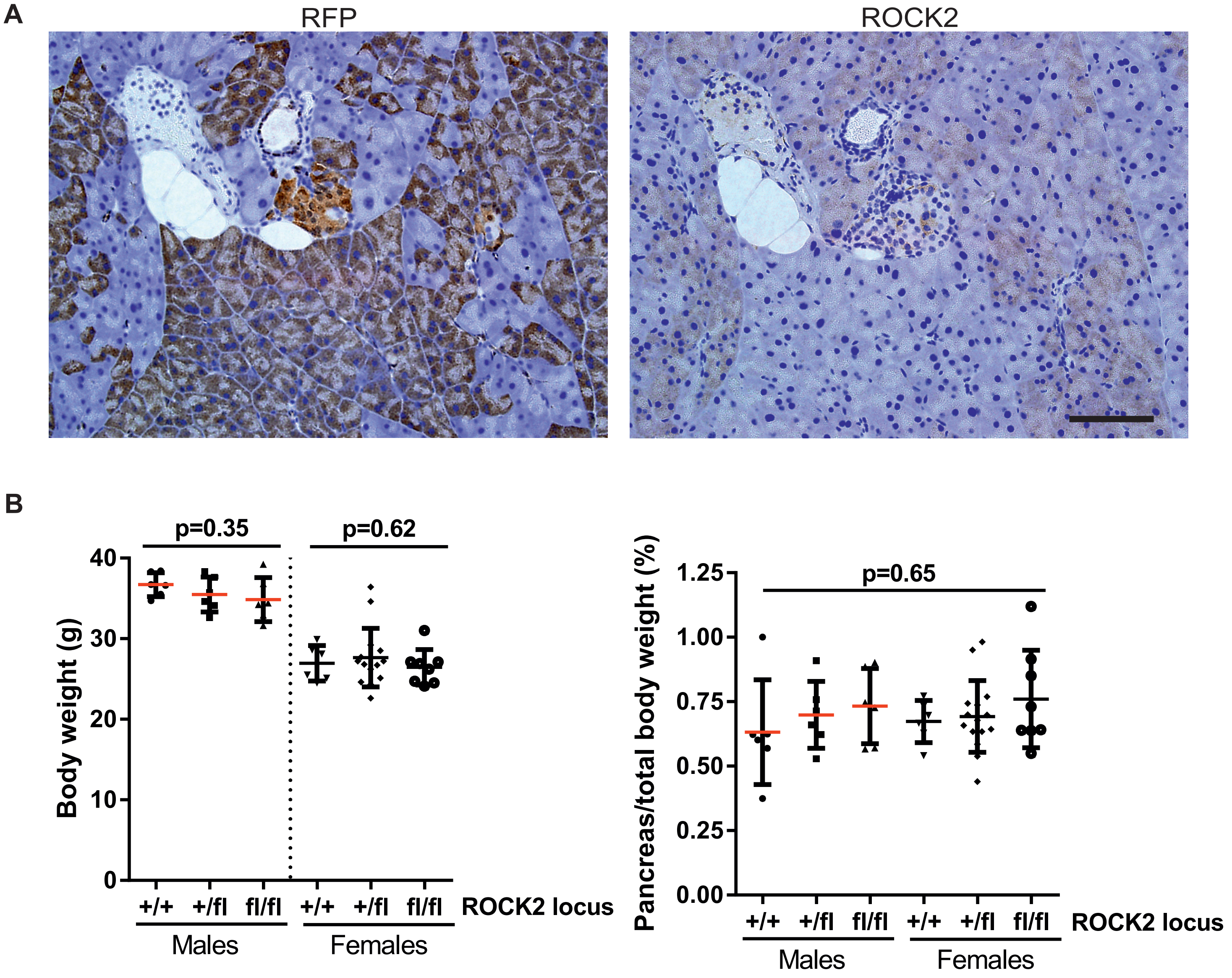

Appendix Fig S1

Appendix Figure S2 - Tissue selective expression of conditionally-regulated ROCK2 (related to Fig 1).

A, B Immunoblot of ROCK2:ER fusion protein in pancreata of 2 *LSL-ROCK2:ER*; *Pdx1-Cre* mice induced by Cre-mediated recombination but not in 2 *LSL-ROCK2:ER* mice (A) and uncropped immunoblot (B).

C Primary acinar cells from *Pdx1-Cre* or *Pdx1-Cre; LSL-ROCK2:ER* mice were cultured with vehicle or 2 μM 4HT, and imaged after 5 days (left, scale bar = 200 μm), or assessed for attachment after 48 h (right). Means ± SEM (n=4), p value by ratio paired t test.

D Primary acinar cells from *Pdx1-Cre; LSL-KRas*$^{G12D/+}$ mice were cultured with vehicle or 10 μM Y27632, and imaged after 48 h (left, scale bar = 200 μm), or assessed for attachment after 48 h (right). Means ± SEM (n=9), p value by ratio paired t test.

E Primary acinar cells from *Pdx1-Cre* mice were cultured with vehicle or 10 μM Y27632, and assessed for attachment after 48 h. Means ± SEM (n=4), p value by ratio paired t test.

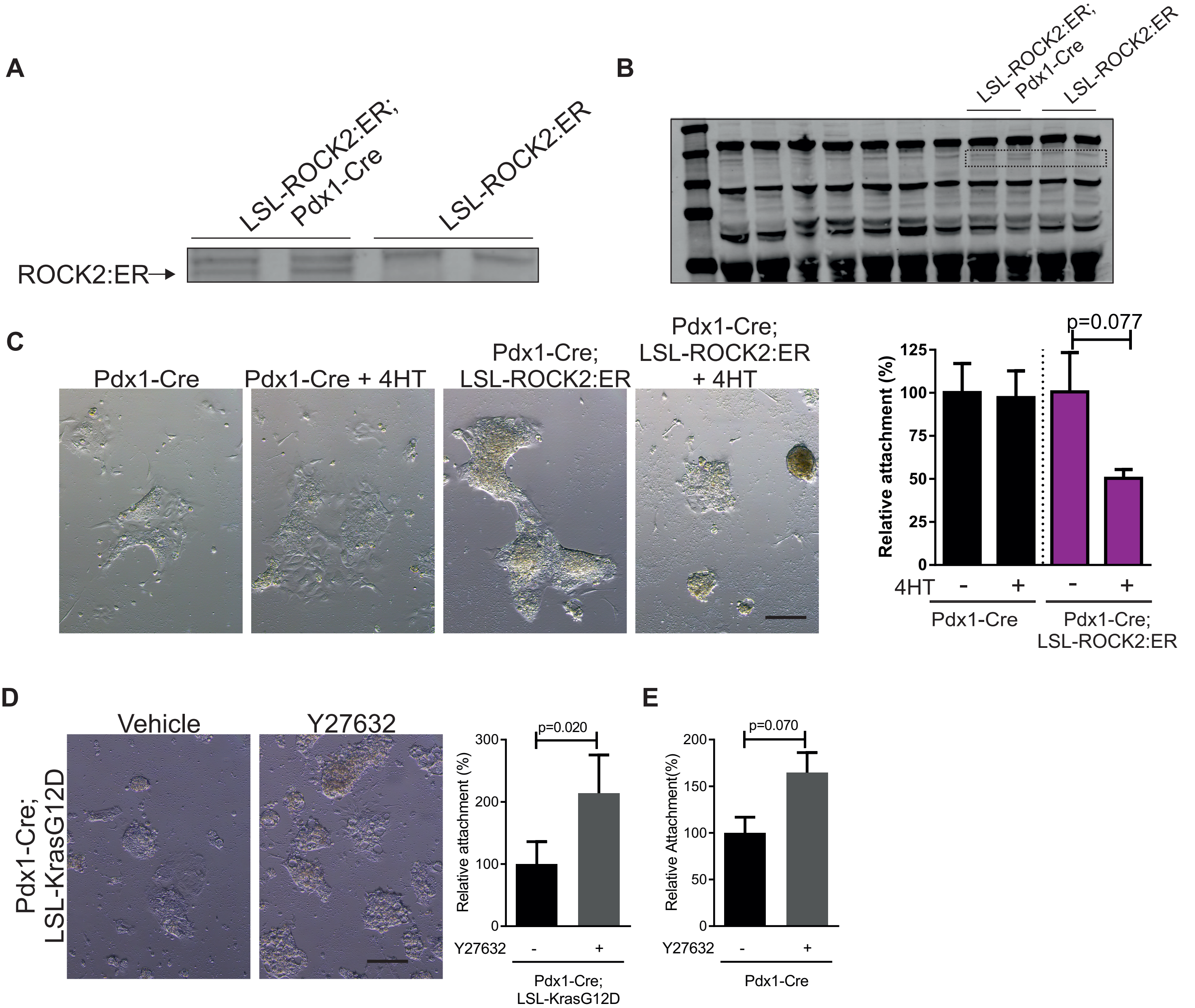

Appendix Fig S2

Appendix Figure S3 - Conditional ROCK activation in KC mice does not alter PanIN numbers (related to Fig 1).

Pdx1-Cre; LSL-KRas$^{G12D/+}$ (KC) or *Pdx1-Cre; LSL-KRas$^{G12D/+}$*; *LSL-ROCK2:ER* (RKC) mice were treated with 1% EtOH vehicle or tamoxifen citrate (100 mg/L) for conditional ROCK activation for 14 days starting at 6 weeks of age. No differences in the incidence of PanINs were observed in 8 week old mice (n=7). Box plots with p-value determined by Kruskal-Wallis test.

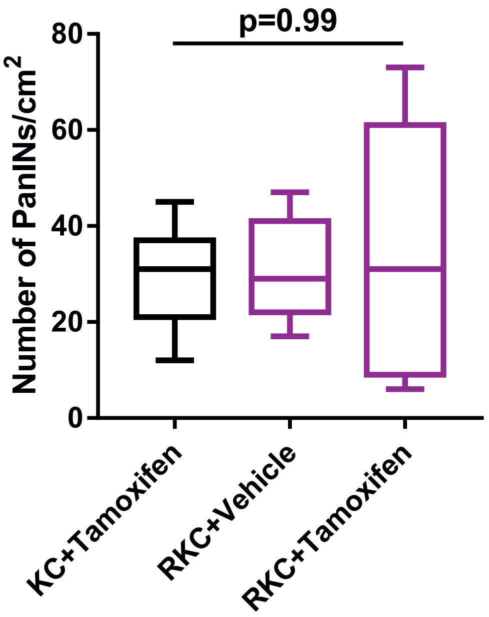

Appendix Fig S3

Appendix Figure S4 - Invasion of KPC or KPflC cells into fibroblast conditioned collagen (related to Fig 2).

H&E stained sections of cell invasion into collagen matrix after 8 days, in the presence (top) or absence of matrix-embedded fibroblasts (bottom). Scale bar = 100 μm. Invasion index of KPC and KPflC cells. Means ± SEM (n=6), p value by unpaired t test.

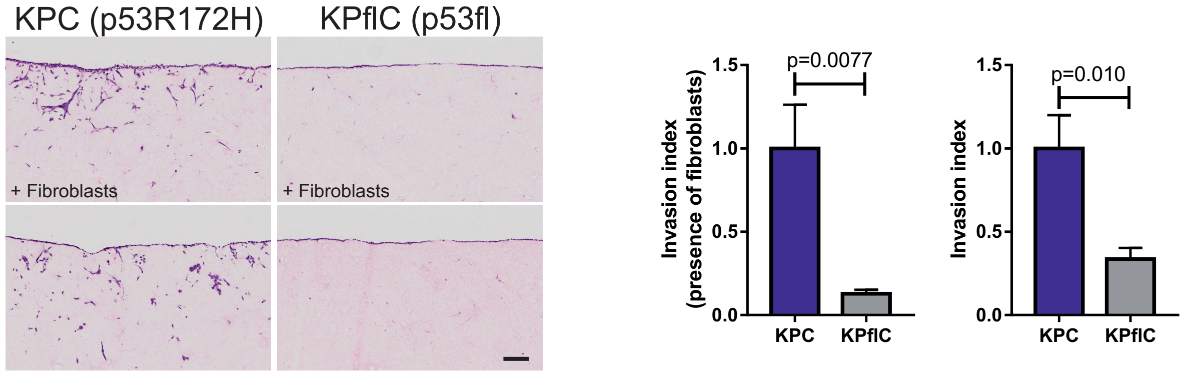

Appendix Fig S4

Appendix Figure S5 - ROCK-induced substrate phosphorylation and changes in cell morphology (related to Fig 2).

A Ratio of phosphorylated MLC (pMLC) to total MLC for KPflC cells expressing indicated ER-fusion proteins as shown in Fig 2C. Cells were left untreated or treated with 4HT in the absence or presence of 1 µM or 10 µM H1152 as indicated. Means ± SEM (n=3).

B KPflC cells expressing indicated ER-fusion proteins, grown as adherent cells and treated with vehicle or 1 µM 4HT for 24 h (scale bar = 50 µm).

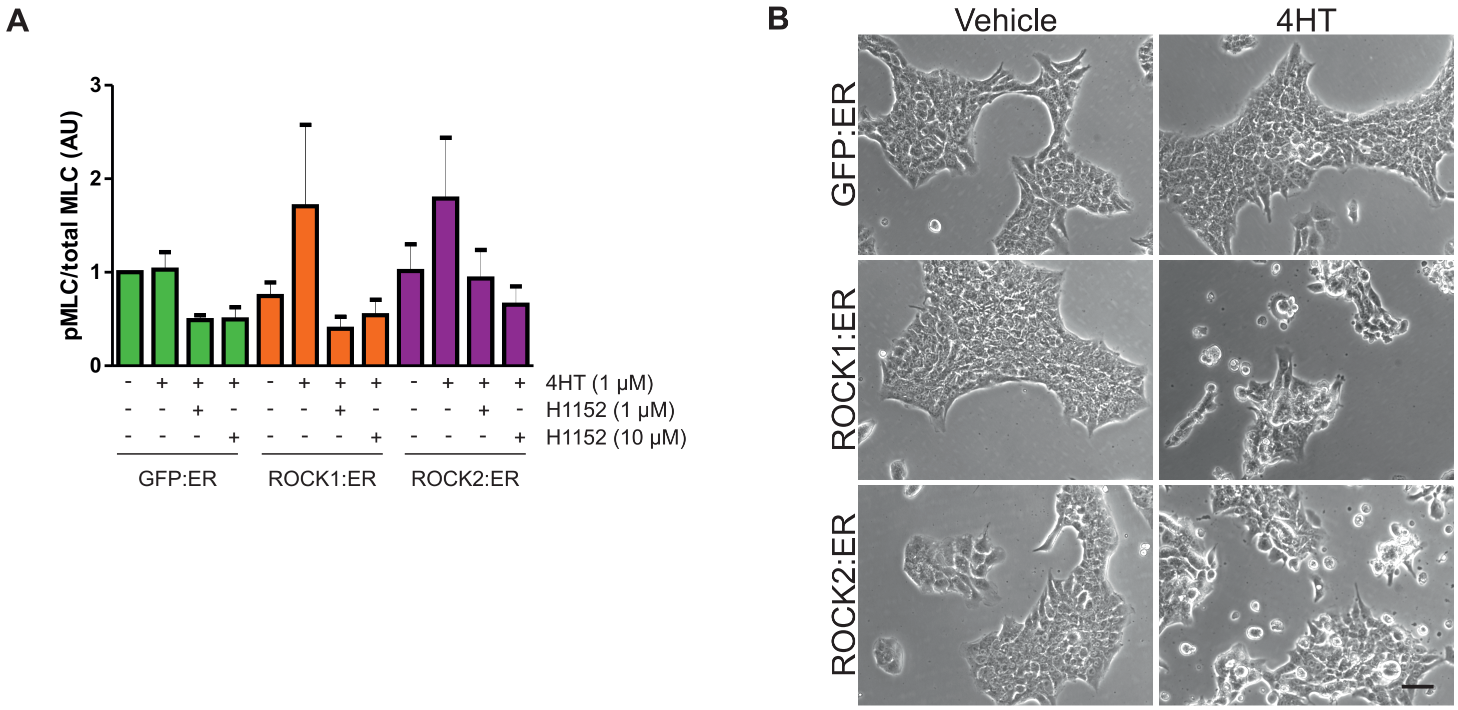

Appendix Fig S5

Appendix Figure S6 - Average RNA sequence reads for indicated collagens with >10 copies detected. (related to Fig 4).

Means ± SEM (n=3) * adjusted p-value <0.05.

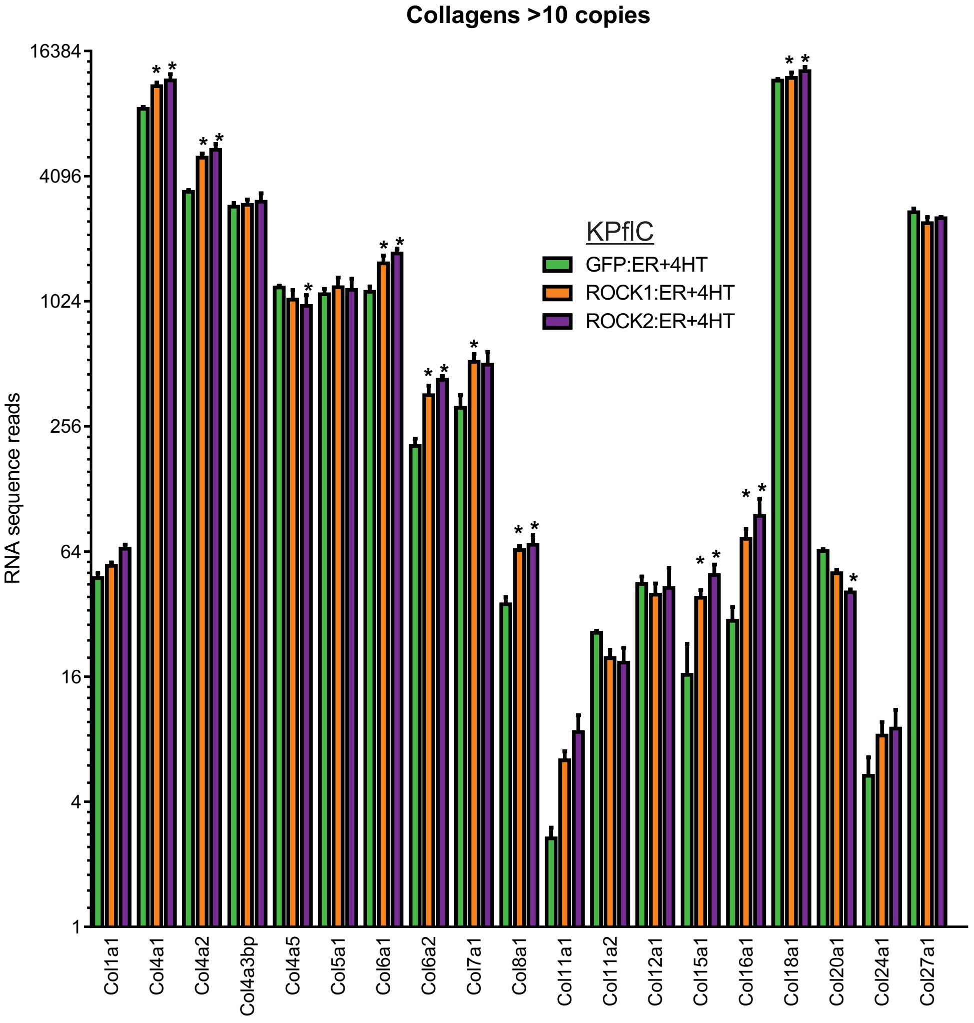

Appendix Fig S6

Appendix Figure S7 - Analysis of KPC mouse tumors with Fasudil ROCK inhibitor treatment (related to Fig 8).

A-E PDAC endpoint tumors were examined for percentages of SMA positive area, Ki67 positive cells, necrotic area, CD3 positive cells and CD31 positive cells as indicated. Box plots with exact p value by Mann-Whitney test.

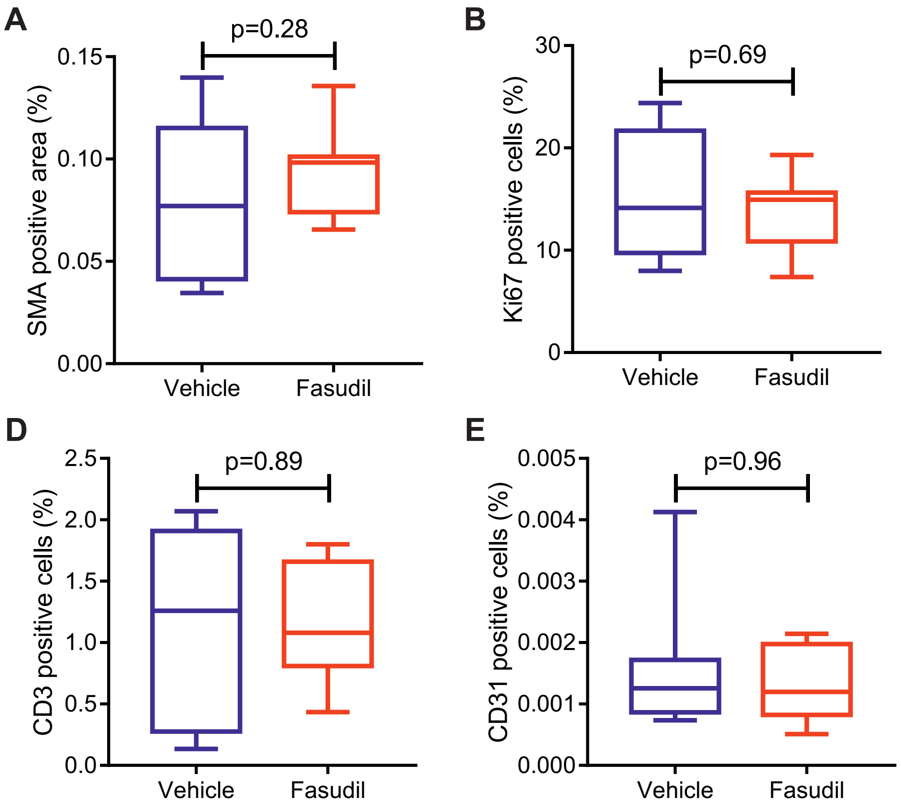

Appendix Fig S7

Appendix Table S1 - ROCK induced changes in gene expression associated with Metacore^{TM} gene process networks.

	Networks	Total in network	p-value	FDR	In Data	Network Objects from Active Data
1	Cell adhesion_Cell-matrix interactions	211	3.551E-08	5.398E-06	37	MMP-12, Galectin-3, ADAM33, ADAM-TS1, COL4A2, ADAM23, Fibulin-2, ITGA6, COL6A1, MMP-13, CD44 (ICD), LAMC2, TIMP3, Stromelysin-2, Aggrecanase-1, LAMB3, Collagen IV, CD44, ITGA3, ADAM-TS15, CD38, Tenascin-C, LAMB1, ITIH2, COL16A1, A1M, ITGB6, CD44 soluble, Aggrecan, ADAM-TS14, LAMR1, TIMP1, Connexin 43, CD44 (EXT), Aggrecanase-2, Versican, TINAG
2	Proteolysis_Connective tissue degradation	119	2.664E-06	2.025E-04	23	MMP-12, Meprin, ADAM33, ADAM-TS1, ADAM23, Meprin A, alpha, MMP-13, TIMP3, ADAM8, Stromelysin-2, Aggrecanase-1, Collagen IV, PLAT (TPA), ADAM-TS15, Tenascin-C, COL16A1, PAI1, PLAU (UPA), Aggrecan, ADAM-TS14, TIMP1, Aggrecanase-2, TINAG
3	Cell adhesion_Integrin-mediated cell-matrix adhesion	214	2.845E-05	1.135E-03	31	Tubulin beta, Talin, Cyclin D1, PI3K cat class IA, Tubulin beta 3, Fibulin-2, ITGA6, Tubulin alpha, Beta-parvin, Filamin A, Caveolin-1, Collagen IV, Tubulin alpha 1A, Lpd, ITGA3, LIMK1, CD53, Tenascin-C, ERM proteins, MyHC, Tcf(Lef), Filamin B (TABP), ITGB6, Zyxin, Tubulin beta 2, PI3K cat class IA (p110-delta), Filamin C, Cyr61, TINAG, Tubulin alpha-4A, Tubulin (in microtubules)
4	Development_Ossification and bone remodeling	157	3.477E-05	1.135E-03	25	ATF-4, GDF11(BMP11), TGF-beta, TIEG, SHH, Filamin A, Ihh, TWIST1, Hedgehog, FGFR2, CSF1, TIEG1, SLC26A2, Spp24, WNT7A, ETS, WNT, BMP4, Annexin II, p38 MAPK, Galpha(s)-specific calcitonin GPCRs, p90Rsk, IBP, Frizzled, Activin
5	Signal transduction_WNT signaling	177	3.732E-05	1.135E-03	27	COX-2 (PTGS2), WNT4, TGF-beta 2, WNT10A, PP2A regulatory, TGF-beta, Cyclin D1, DLX2, Adenylate cyclase type I, ERK3, E2F2, p38beta (MAPK11), ESR1 (nuclear), CD44, WNT7B, Fra-1, DAAM1, Tcf(Lef), WNT7A, Cyclin E, WNT, SLUG, p38 MAPK, Adenylate cyclase, TCF7L2 (TCF4), Frizzled, IP3 receptor
6	Cytoskeleton_Regulation of cytoskeleton rearrangement	183	6.772E-05	1.716E-03	27	Ankyrin-G, Plectin 1, Tubulin beta, Talin, SPTBN(spectrin1-4), Advillin (p92), Tubulin beta 3, Galpha(i)-specific amine GPCRs, RhoF (Rif), Tubulin alpha, Filamin A, VAV-3, RHO6, Tubulin alpha 1A, CD43, CD44, LIMK1, ERM proteins, MyHC, Filamin B (TABP), Beta-fodrin, Zyxin, Tubulin beta 2, Filamin C, WaspIP, Protein kinase G, Tubulin (in microtubules)
7	Proliferation_Negative regulation of cell proliferation	184	1.897E-04	4.120E-03	26	COX-2 (PTGS2), GDF11(BMP11), GAB2, Galpha(i)-specific peptide GPCRs, FGFR3, ADAM-TS1, TGF-

						beta, Cyclin D1, PI3K cat class IA, Amphiregulin, Calgizzarin, VEGFR-2, GRO-1, OSMR, BTG2, p21, IBP5, PI3K reg class IB (p101), ETS1, Cyclin E, Sestrin 1, IBP, MAD4, IL6RA, C/EBPalpha, IBP3
8	Blood coagulation	94	4.137E-04	7.860E-03	16	Galpha(q)-specific nucleotide-like GPCRs, MSP, Thrombomodulin, PAR1, Caveolin-1, Collagen IV, Protein C receptor (endothelial), Tissue factor, PLAT (TPA), Protein S, Protein C, PAR2, PAI1, Adenosine A2a receptor, SERPINF2, PLAU (UPA)
9	Development_Blood vessel morphogenesis	228	5.137E-04	8.675E-03	29	Neuropilin-2, Galpha(i)-specific peptide GPCRs, Galpha(q)-specific nucleotide-like GPCRs, PI3K cat class IA, Galpha(i)-specific amine GPCRs, Amphiregulin, VEGFR-2, PAR1, Galpha(q)-specific peptide GPCRs, ALDOA, Transferrin, Galpha(s)-specific nucleotide-like GPCRs, LDHA, Alpha-1B adrenergic receptor, PGAR, PLGF, PDE3B, PDE, PLAT (TPA), Neuregulin 1, Epiregulin, BMP4, Galpha(q)-specific amine GPCRs, Adenosine A2a receptor, PGK1, Neuropilin-1, Protein kinase G, Cyr61, FGF1
10	Cell adhesion_Platelet aggregation	158	7.154E-04	1.087E-02	22	COX-2 (PTGS2), GAB2, Talin, PI3K cat class IA, PAR1, Adenylate cyclase type I, PLA2G7, Filamin A, VAV-3, Alpha-2A adrenergic receptor, Collagen IV, PLA2, MyHC, Ca-ATPase3, THAS, P-selectin, ENTPD2-alpha, Gab, Adenylate cyclase, PI3K cat class IA (p110-delta), IP3 receptor, Adenylate cyclase type VII
11	Proteolysis_ECM remodeling	85	1.306E-03	1.678E-02	14	MMP-12, ADAM33, MMP-13, TIMP3, Stromelysin-2, Aggrecanase-1, Collagen IV, PLAT (TPA), Tenascin-C, PAI1, PLAU (UPA), Aggrecan, ADAM-TS14, TIMP1
12	Cytoskeleton_Actin filaments	176	1.324E-03	1.678E-02	23	Ankyrin-G, Plectin 1, Talin, SPTBN(spectrin1-4), MYH10, Filamin A, CD44, Myosin I, Troponin T, cardiac, LIMK1, DAAM1, ERM proteins, MyHC, Filamin B (TABP), Annexin II, NEBL, Beta-fodrin, Zyxin, Tropomyosin-4, Filamin C, Tropomyosin, WaspIP, Protein kinase G
13	Cell adhesion_Cell junctions	162	2.340E-03	2.735E-02	21	GJC1, Tubulin beta, Nectin-2, JAM2, ZO-3, PI3K cat class IA, ATP1B1, Tubulin alpha, Desmocollin 3, WNK4, Plakophilin 1, Connexin 45, Caveolin-1, Tcf(Lef), DSC2, Beta-fodrin, Connexin 31, Claudin-7, Connexin 43, SIP1 (ZFHX1B), Tubulin (in microtubules)
14	Development_EMT_Regulation of epithelial-to-mesenchymal transition	225	3.882E-03	4.215E-02	26	COX-2 (PTGS2), PDGF-A, GAB2, 4E-BP1, TGF-beta 2, Nestin, TGF-beta, PI3K cat class IA, HMGA2, PDGF-B, TWIST1, FGFR2, ESR1 (nuclear), Keratin 14, ETS1, Cyclin E, WNT, BMP4, SLUG, p38 MAPK, PAI1, SOX9, p90Rsk, Frizzled, SIP1 (ZFHX1B), FGF1
15	Development_Neurogenesis_Axonal guidance	230	5.208E-03	5.277E-02	26	Neuropilin-2, Ephrin-A receptor 7, MYH10, ADAM23, PI3K cat class IA, Ephrin-A receptors, APOE, FMR2, DCAMKL1, Adenylate cyclase type I, CRMP4, RHO6, DARPP-32,

						Semaphorin 7A, LIMK1, APOER2, ERM proteins, MyHC, BDNF, Ephrin-A receptor 2, Selenoprotein P, Neuropilin-1, IP3 receptor, MIR (Idol), Tubulin (in microtubules), Plexin A4
16	Cell adhesion_Cadherins	180	8.076E-03	7.672E-02	21	WNT4, Galectin-3, PTP-2, PTPRF (LAR), Nectin-2, WNT10A, PTPR-mu, PI3K cat class IA, FXYD5, Desmocollin 3, Plakophilin 1, ITGA3, PDZK3, VLDLR, Tcf(Lef), WNT7A, WNT, DSC2, LI-cadherin, Frizzled, Tubulin (in microtubules)
17	Reproduction_FSH-beta signaling pathway	160	9.538E-03	8.528E-02	19	4E-BP1, TGF-beta 2, FSRP, PP2A regulatory, PI3K cat class IA, p90RSK3(RPS6KA2), Activin beta A, DUSP5, Adenylate cyclase type I, PEA3, Epiregulin, BMP4, p38 MAPK, Adenylate cyclase, p90Rsk, IBP, IP3 receptor, Cyclin D, IBP3
18	Cardiac development_Wnt_beta-catenin, Notch, VEGF, IP3 and integrin signaling	150	1.033E-02	8.727E-02	18	Neuropilin-2, ID1, GLI-3R, CRLR, VEGFR-2, GLI-3, Connexin 45, Alpha-1B adrenergic receptor, Troponin T, cardiac, MyHC, Tcf(Lef), HHEX (PRH), WNT, Galpha(s)-specific calcitonin GPCRs, Frizzled, Connexin 43, IP3 receptor, Versican
19	Development_Neurogenesis_Synaptogenesis	180	1.592E-02	1.274E-01	20	Homer, Synaptotagmin, X11, Neuregulin 4, Synapsin I, Semaphorin 4F, APOE, nAChR alpha, FGFR2, Synaptotagmin XI, Neuregulin 2, APOER2, Neuregulin 1, WNT7A, FGFR4, WNT, BDNF, DLGAP1 (GKAP), Semaphorin 4G, Frizzled
20	Cytoskeleton_Intermediate filaments	81	1.762E-02	1.339E-01	11	Plectin 1, Tubulin beta, Nestin, Tubulin alpha, Plakophilin 1, Desmuslin, Keratin 14, ZNF239, Tubulin beta 2, Lamin A/C, Tubulin (in microtubules)
21	Chemotaxis	137	1.916E-02	1.387E-01	16	GRO-2, Galpha(i)-specific peptide GPCRs, PI3K cat class IA, CXCL16, GRO-1, Galpha(q)-specific peptide GPCRs, CCL13, CD43, CD44, Fra-1, CCL2, GRO-3, p38 MAPK, C3aR, PLAU (UPA), Cyr61
22	Proliferation_Positive regulation cell proliferation	221	2.097E-02	1.442E-01	23	COX-2 (PTGS2), GAB2, Galpha(i)-specific peptide GPCRs, Glypican-1, Cyclin D1, PI3K cat class IA, Galpha(i)-specific amine GPCRs, SHH, FGFR2, Alpha-2A adrenergic receptor, CSF1, p21, Fra-1, PLGF, Alpha-1D adrenergic receptor, Cyclin E, GM-CSF, N-Myc, Neuropilin-1, PI3K cat class IA (p110-delta), TIMP1, LIF, LIFR
23	Cell adhesion_Platelet-endothelium-leucocyte interactions	174	2.182E-02	1.442E-01	19	PDGF-A, TGF-beta 2, JAM2, TGF-beta, Thrombomodulin, VEGFR-2, MMP-13, PDGF-B, Collagen IV, CD44, CCL2, PLAT (TPA), VLDLR, Protein S, Protein C, P-selectin, PAI1, PLAU (UPA), Cyr61
24	Immune response_Th17-derived cytokines	98	2.850E-02	1.787E-01	12	COX-2 (PTGS2), C/EBP, PI3K cat class IA, PGES, GRO-1, MMP-13, Calgranulin A, CCL2, Calgranulin B, p38 MAPK, GM-CSF, TIMP1
25	Development_Cartilage development	66	2.955E-02	1.787E-01	9	TR-alpha, Activin beta A, MMP-13, p38beta (MAPK11), Ihh, Aggrecanase-1, BMP4, SOX9, Aggrecan
26	Development_Hedgehog signaling	254	3.057E-02	1.787E-01	25	ID1, LRP2 (Megalin), Cyclin D1, GLI-3R, Activin beta A, DLX2, DHH, SHH, Adenylate cyclase type I, Filamin A, p38beta (MAPK11), Ihh, Hedgehog, FGFR2, GLI-3, p21,

						ROR-alpha, PI3K reg class IB (p101), BMP4, N-Myc, Adenylate cyclase, SOX9, TCF7L2 (TCF4), Frizzled, Adenylate cyclase type VII
27	Apoptosis_Anti-apoptosis mediated by external signals via NF-kB	111	3.209E-02	1.806E-01	13	COX-2 (PTGS2), PDGF-A, PP2A regulatory, PI3K cat class IA, VEGFR-2, PDGF-B, Bcl-3, APRIL(TNFSF13), PI3K reg class IB (p101), GM-CSF, Adenylate cyclase, p90Rsk, FN14(TNFRSF12A)
28	Cell adhesion_Amyloid proteins	195	3.433E-02	1.864E-01	20	PAK3, WNT4, BACE2, ADAM33, TGF-beta 2, X11, WNT10A, Filamin A, Caveolin-1, Collagen IV, WNT7B, PDZK3, LIMK1, Tcf(Lef), WNT7A, Filamin B (TABP), WNT, Frizzled, Connexin 43, Tubulin (in microtubules)
29	Inflammation_IL-13 signaling pathway	91	3.808E-02	1.953E-01	11	IL1RN, Adenylate cyclase type I, IL13RA1, DCOR, CCL2, Tenascin-C, HSD3B1, FOXJ1, PI3K cat class IA (p110-delta), IP3 receptor, SOCS1
30	Reproduction_Feeding and Neurohormone signaling	211	4.058E-02	1.953E-01	21	4E-BP1, TGF-beta 2, Galpha(i)-specific peptide GPCRs, LRP2 (Megalin), PP2A regulatory, TGF-beta, MSP, PI3K cat class IA, Galpha(q)-specific peptide GPCRs, Adenylate cyclase type I, FCGRT, PEA3, CD44, DCOR, ITGA3, Cyclin E, PAI1, Pim-1, SOX9, IBP3, SOCS1
31	Signal transduction_NOTCH signaling	236	4.074E-02	1.953E-01	23	PDGF-A, WNT4, 4E-BP1, TGF-beta 2, WNT10A, TGF-beta, GLUT1, Cyclin D1, PI3K cat class IA, Amphiregulin, PDGF-B, p38beta (MAPK11), p21, CD44, WNT7B, Neuregulin 1, WNT7A, Epiregulin, FZD7, WNT, p38 MAPK, Frizzled, PI3K cat class IA (p110-delta)
32	Cytoskeleton_Cytoplasmic microtubules	115	4.112E-02	1.953E-01	13	Tubulin beta, X11, Dynein, axonemal, heavy chains, Tubulin beta 3, RhoF (Rif), Tubulin alpha, MAP6, Tubulin alpha 1A, MAPRE2(RP1), TUBGCP2, DLGAP1 (GKAP), HOOK2, Tubulin (in microtubules)
33	Reproduction_Progesterone signaling	214	4.611E-02	2.068E-01	21	COX-2 (PTGS2), ESR1 (membrane), MIF, ADAM-TS1, PI3K cat class IA, PDE3, Galpha(q)-specific peptide GPCRs, Adenylate cyclase type I, JDP2, ESR1 (nuclear), PLA2, DHB4, PDE3B, Tcf(Lef), WNT, Adenylate cyclase, Galpha(s)-specific calcitonin GPCRs, p90Rsk, Frizzled, IP3 receptor, Tubulin (in microtubules)
34	Cardiac development_BMP_TGF_beta_signaling	117	4.626E-02	2.068E-01	13	TGF-beta 2, ID1, MYH10, GLI-3R, GLI-3, ESR1 (nuclear), Activin A, Troponin T, cardiac, MyHC, Troponin C, cardiac, BMP4, SLUG, SOX9
35	Inflammation_Amphoterin signaling	118	4.898E-02	2.127E-01	13	PI3K cat class IA, MMP-13, IL1RN, p38beta (MAPK11), Calgranulin A, CCL2, LIMK1, PLAT (TPA), MyHC, Calgranulin B, p38 MAPK, PAI1, PI3K cat class IA (p110-delta)

Appendix Table S2 - RNA sequencing reads for Mmp and Timp transcripts.

								RNA sequence reads								
	symbol	D2_log2FoldChange(ROCK1_4HT_vs_GFP_4HT)	D2_pvalue(ROCK1_4HT_vs_GFP_4HT)	D2_padj(ROCK1_4HT_vs_GFP_4HT)	D2_log2FoldChange(ROCK2_4HT_vs_GFP_4HT)	D2_pvalue(ROCK2_4HT_vs_GFP_4HT)	D2_padj(ROCK2_4HT_vs_GFP_4HT)	GFP_4HT_1	GFP_4HT_2	GFP_4HT_3	ROCK1_4HT_1	ROCK1_4HT_2	ROCK1_4HT_3	ROCK2_4HT_1	ROCK2_4HT_2	ROCK2_4HT_3
Collagenases	**Mmp1a**	NA	NA	NA	NA	NA	NA	0	0	0	0	0	0	0	0	0
	**Mmp1b**	NA	NA	NA	NA	NA	NA	0	0	0	0	0	0	0	0	0
	**Mmp8**	NA	NA	NA	NA	NA	NA	0	0	0	0	0	0	0	0	0
	**Mmp13**	3.405297	2.55E-28	6.06E-26	3.70139	2.94E-33	6.37E-31	50	46	49	476	679	363	530	959	591
Gelatinases	**Mmp2**	0.003215	0.993782	NA	0.359147	0.397855	NA	0	0	0	0	0	0	0	1	0
	**Mmp9**	0.03459	0.967397	NA	1.39613	0.106042	NA	1	0	0	0	0	1	1	5	1
Stromelysins	**Mmp3**	2.321984	0.002659	NA	2.130918	0.005915	NA	0	2	0	4	11	4	4	9	5
	**Mmp10**	4.456138	2.11E-22	2.95E-20	4.952517	1.52E-27	2.19E-25	3	6	2	75	146	69	123	227	108
	**Mmp11**	-0.06396	0.65525	0.774096	-0.17104	0.232381	0.386352	542	541	649	386	494	653	408	592	544
MT-MMPs	**Mmp14**	0.19228	0.052914	0.130848	0.091522	0.356834	0.525506	3236	2977	3810	3214	3245	4117	3354	3633	3668
	**Mmp15**	-0.32209	0.001578	0.007868	-0.34341	0.000745	0.003808	2716	2475	2811	1981	1702	2214	2211	1976	2070
	**Mmp16**	-0.38359	0.379612	NA	-0.38704	0.379023	NA	0	1	0	0	0	0	0	0	0
	**Mmp17**	-0.23058	0.624187	NA	-0.57807	0.227735	0.381344	11	10	19	6	14	11	7	12	7
	**Mmp24**	3.831482	2.25E-08	NA	3.769133	3.68E-08	5.74E-07	0	1	0	17	24	10	13	28	13
	**Mmp25**	0.003561	0.993347	NA	0.388659	0.377848	NA	0	0	0	0	0	0	0	0	1
Matrilysin	**Mmp7**	NA	NA	NA	NA	NA	NA	0	0	0	0	0	0	0	0	0
Others	**Mmp12**	0.003803	0.993036	NA	0.000106	0.999807	NA	0	0	0	0	0	0	0	0	0
	**Mmp19**	0.75619	0.053251	0.131397	0.938257	0.014749	0.045962	14	13	13	12	32	20	19	35	26
	**Mmp20**	NA	NA	NA	NA	NA	NA	0	0	0	0	0	0	0	0	0
	**Mmp21**	-0.37722	0.383874	NA	-0.38059	0.383298	NA	1	0	0	0	0	0	0	0	0
	**Mmp23**	-1.08272	8.24E-05	0.000657	-0.60269	0.021261	0.061806	62	64	80	38	13	37	36	55	44
	**Mmp27**	-0.74639	0.309149	NA	-0.02728	0.971456	NA	1	0	1	0	0	0	0	2	0
	**Mmp28**	0.077473	0.489099	0.641417	-0.06304	0.573828	0.718039	623	519	551	534	488	625	485	531	600
Inhibitors	**Timp1**	0.753121	1.04E-09	2.54E-08	1.006326	1.37E-16	7.25E-15	247	220	252	318	429	370	476	506	464
	**Timp2**	-0.11704	0.017681	0.05635	-0.10203	0.038417	0.099637	13329	12456	13380	10343	10826	12100	11168	12965	12334
	**Timp3**	0.736928	3.72E-13	1.66E-11	0.646805	1.78E-10	4.18E-09	1345	1118	1004	1719	1672	1939	1538	1984	1921
	**Timp4**	-2.79873	7.79E-05	NA	-2.03629	0.001699	NA	10	9	13	0	1	1	2	4	0

Appendix Table S3 - RNA sequencing reads for collagen transcripts.

							RNA sequence reads								
symbol	D2_log2FoldChange(ROCK1_4HT_vs_GFP_4HT)	D2_pvalue(ROCK1_4HT_vs_GFP_4HT)	D2_padj(ROCK1_4HT_vs_GFP_4HT)	D2_log2FoldChange(ROCK2_4HT_vs_GFP_4HT)	D2_pvalue(ROCK2_4HT_vs_GFP_4HT)	D2_padj(ROCK2_4HT_vs_GFP_4HT)	GFP_4HT_1	GFP_4HT_2	GFP_4HT_3	ROCK1_4HT_1	ROCK1_4HT_2	ROCK1_4HT_3	ROCK2_4HT_1	ROCK2_4HT_2	ROCK2_4HT_3
**Col1a1**	0.312066	0.15701	0.291354	0.465923	0.030863	0.083734	53	45	45	51	55	58	70	68	60
**Col1a2**	0.018414	0.979364	NA	1.512879	0.04886	NA	0	0	0	0	0	0	2	1	1
**Col2a1**	0.415513	0.630897	NA	-0.01121	0.989603	NA	1	0	0	0	1	1	0	1	0
**Col3a1**	0.415347	0.482824	NA	0.406062	0.495073	NA	0	0	0	0	0	1	0	0	1
**Col4a1**	0.478588	2.31E-13	1.06E-11	0.45232	4.20E-12	1.25E-10	8556	8992	8357	10570	10714	11991	10231	12914	12375
**Col4a2**	0.663892	3.44E-23	5.02E-21	0.667922	1.66E-23	1.70E-21	3380	3562	3392	4756	4852	5492	4765	5881	5800
**Col4a3**	-0.15392	0.809322	NA	0.082687	0.894652	NA	5	7	4	4	4	5	7	5	5
**Col4a3bp**	0.147223	0.10393	0.217937	0.076039	0.40079	0.567474	3131	2736	2886	2834	2767	3336	2480	3322	3443
**Col4a4**	-0.18678	0.82894	NA	-0.85667	0.317038	NA	0	3	1	0	1	2	0	1	0
**Col4a5**	-0.08187	0.509388	0.658514	-0.30197	0.015111	0.04692	1202	1154	1228	866	1003	1259	726	1088	1103
**Col4a6**	0.335089	0.701462	NA	0.004966	0.995471	NA	1	1	0	1	0	2	0	0	2
**Col5a1**	0.222949	0.132179	0.257836	0.067658	0.647867	0.776333	995	1094	1228	1035	1083	1467	892	1173	1417
**Col5a2**	0.005609	0.990904	NA	0.00014	0.999775	NA	0	0	0	0	0	0	0	0	0
**Col5a3**	-1.12081	0.117833	NA	-0.48486	0.476544	NA	5	7	3	0	2	3	3	4	3
**Col6a1**	0.568581	2.02E-08	3.97E-07	0.609569	1.66E-09	3.36E-08	1113	1027	1270	1324	1546	1803	1553	1875	1784
**Col6a2**	0.921556	5.28E-10	1.35E-08	1.055604	7.92E-13	2.63E-11	206	176	236	348	300	438	395	441	453
**Col6a3**	1.340891	0.098938	NA	1.415439	0.080235	NA	0	0	3	4	5	2	1	6	6
**Col6a4**	NA	NA	NA	NA	NA	NA	0	0	0	0	0	0	0	0	0
**Col6a5**	NA	NA	NA	NA	NA	NA	0	0	0	0	0	0	0	0	0
**Col6a6**	0.421594	0.351879	NA	0.000109	0.999804	NA	0	0	0	0	1	0	0	0	0
**Col7a1**	0.829486	0.004379	0.018278	0.682067	0.019145	0.056802	336	223	384	562	430	577	447	415	659
**Col8a1**	0.968036	0.000102	0.000786	0.936309	0.000159	0.001017	39	38	30	65	70	60	59	63	85
**Col8a2**	-0.36364	0.675884	NA	-0.41646	0.631878	NA	2	1	2	2	1	0	2	0	1
**Col9a1**	NA	NA	NA	NA	NA	NA	0	0	0	0	0	0	0	0	0
**Col9a2**	0.672602	0.384395	NA	-0.4569	0.537239	NA	0	0	1	0	2	1	0	0	0
**Col9a3**	0.200064	0.74867	NA	0.50452	0.406033	NA	4	3	5	3	6	4	6	6	6
**Col10a1**	0.745259	0.270492	NA	0.413016	0.537771	NA	0	0	0	2	0	0	1	0	0
**Col11a1**	1.132016	0.071311	NA	1.424773	0.020579	NA	2	3	3	5	7	7	6	12	8
**Col11a2**	-0.27371	0.408348	0.569186	-0.46562	0.162588	0.297879	25	27	26	22	16	21	17	25	14
**Col12a1**	-0.05517	0.850216	0.907857	-0.0724	0.803088	0.881337	49	48	37	35	34	50	22	47	59
**Col13a1**	0.003215	0.993782	NA	0.359147	0.397855	NA	0	0	0	0	0	0	0	1	0
**Col14a1**	-0.12439	0.878328	NA	-0.20902	0.79673	NA	1	2	2	1	2	1	0	1	3
**Col15a1**	1.297059	0.000283	0.001857	1.521421	1.61E-05	0.000138	28	5	16	40	43	32	39	60	49
**Col16a1**	1.38897	7.29E-07	1.03E-05	1.620727	4.94E-09	9.22E-08	21	38	30	57	79	85	56	120	109
**Col17a1**	-0.09078	0.91364	NA	-0.51593	0.540526	NA	1	2	4	2	0	4	2	1	1
**Col18a1**	0.160285	0.010168	0.036336	0.152742	0.01427	0.044695	11749	11481	12154	11385	11448	13637	12204	14244	12890
**Col19a1**	NA	NA	NA	NA	NA	NA	0	0	0	0	0	0	0	0	0
**Col20a1**	-0.23053	0.271841	0.431818	-0.65015	0.002715	0.011386	65	66	62	54	47	50	38	42	42
**Col22a1**	NA	NA	NA	NA	NA	NA	0	0	0	0	0	0	0	0	0
**Col23a1**	0.034988	0.967894	NA	0.005451	0.995001	NA	1	1	0	0	0	2	0	0	2
**Col24a1**	0.67632	0.219197	NA	0.68377	0.210801	0.360879	3	7	6	11	7	7	12	5	10
**Col25a1**	0.003215	0.993782	NA	0.359147	0.397855	NA	0	0	0	0	0	0	0	1	0
**Col26a1**	0.947476	0.277441	NA	0.876831	0.314868	NA	1	0	0	1	2	1	2	2	0
**Col27a1**	-0.05436	0.570746	0.707291	-0.08741	0.36151	0.530112	2974	2630	2634	2654	2109	2534	2561	2517	2635
**Col28a1**	-0.78436	0.260309	NA	-0.3594	0.617	NA	1	0	1	0	0	0	0	1	0

Appendix Table S4 - Clinical information data provided by US Biomax for Pancreas cancer tissue array (PA961c).

catalognum	position	sex	age	organ	pathology	grade	stage	tnm	type
PA961c	A1	F	67	Pancreas	Duct adenocarcinoma	1	III	T3N1bM0	Malignant
PA961c	A2	F	48	Pancreas	Duct adenocarcinoma	1	IIA	T3N0M0	Malignant
PA961c	A3	F	47	Pancreas	Duct adenocarcinoma	1	IIA	T3N0M0	Malignant
PA961c	A4	M	59	Pancreas	Duct adenocarcinoma	1	IIB	T2N1M0	Malignant
PA961c	A5	F	44	Pancreas	Adenocarcinoma (sparse)	-	IIB	T3N1M0	Malignant
PA961c	A6	M	34	Pancreas	Duct adenocarcinoma	1	IIA	T3N0M0	Malignant
PA961c	A7	M	49	Pancreas	Duct adenocarcinoma	1	IB	T2N0M0	Malignant
PA961c	A8	M	56	Pancreas	Duct adenocarcinoma	1	IB	T2N0M0	Malignant
PA961c	A9	M	49	Pancreas	Duct adenocarcinoma	1	IB	T2N0M0	Malignant
PA961c	A10	M	67	Pancreas	Duct adenocarcinoma	1	IB	T2N0M0	Malignant
PA961c	A11	M	48	Pancreas	Duct adenocarcinoma	1	IB	T2N0M0	Malignant
PA961c	A12	F	56	Pancreas	Duct adenocarcinoma	-	IB	T2N0M0	Malignant
PA961c	B1	F	58	Pancreas	Duct adenocarcinoma	1	IIB	T2N1M0	Malignant
PA961c	B2	M	52	Pancreas	Duct adenocarcinoma	1	IIB	T3N1M0	Malignant
PA961c	B3	F	51	Pancreas	Duct adenocarcinoma	-	IIA	T3N0M0	Malignant
PA961c	B4	M	55	Pancreas	Duct adenocarcinoma	1	IIA	T3N0M0	Malignant
PA961c	B5	F	62	Pancreas	Duct adenocarcinoma with necrosis	2	III	T4N0M0	Malignant
PA961c	B6	M	56	Pancreas	Duct adenocarcinoma	2	IB	T2N0M0	Malignant
PA961c	B7	M	60	Pancreas	Duct adenocarcinoma	2	IA	T2N0M0	Malignant
PA961c	B8	M	52	Pancreas	Duct adenocarcinoma	-	IIA	T3N0M0	Malignant
PA961c	B9	M	42	Pancreas	Hyperplasia of duct epithelium	-	IB	T2N0M0	Malignant
PA961c	B10	M	54	Pancreas	Duct adenocarcinoma	-	IIA	T3N0M0	Malignant
PA961c	B11	F	51	Pancreas	Duct adenocarcinoma	1	IIA	T3N0M0	Malignant
PA961c	B12	F	54	Pancreas	Duct adenocarcinoma	2	IB	T2N0M0	Malignant
PA961c	C1	M	54	Pancreas	Duct adenocarcinoma	2	IIA	T3N0M0	Malignant
PA961c	C2	M	39	Pancreas	Duct adenocarcinoma	2	IIA	T3N0M0	Malignant
PA961c	C3	F	41	Pancreas	Duct adenocarcinoma	-	IIA	T3N0M0	Malignant
PA961c	C4	F	68	Pancreas	Duct adenocarcinoma	2	IIA	T3N0M0	Malignant
PA961c	C5	F	44	Pancreas	Duct adenocarcinoma	2	IIB	T3N1M0	Malignant
PA961c	C6	M	42	Pancreas	Duct adenocarcinoma	1	IIA	T3N0M0	Malignant
PA961c	C7	M	53	Pancreas	Duct adenocarcinoma	2	IB	T2N0M0	Malignant
PA961c	C8	M	51	Pancreas	Duct adenocarcinoma	1	IIA	T3N0M0	Malignant

PA961c	C9	M	48	Pancreas	Duct adenocarcinoma	1	IIA	T3N0M0	Malignant
PA961c	C10	M	57	Pancreas	Duct adenocarcinoma	-	IIA	T3N0M0	Malignant
PA961c	C11	F	64	Pancreas	Duct adenocarcinoma	1	IIA	T3N0M0	Malignant
PA961c	C12	M	49	Pancreas	Duct adenocarcinoma	2	IIA	T3N0M0	Malignant
PA961c	D1	M	72	Pancreas	Duct adenocarcinoma	1	IIA	T3N0M0	Malignant
PA961c	D2	F	72	Pancreas	Duct adenocarcinoma	2	IIA	T3N0M0	Malignant
PA961c	D3	M	59	Pancreas	Duct adenocarcinoma	2	IIA	T3N0M0	Malignant
PA961c	D4	F	45	Pancreas	Duct adenocarcinoma	2	III	T3N2M0	Malignant
PA961c	D5	F	60	Pancreas	Duct adenocarcinoma	2	IV	T2N1M1	Malignant
PA961c	D6	M	52	Pancreas	Chronic inflammation of pancreas tissue	-	IA	T1N0M0	Malignant
PA961c	D7	F	44	Pancreas	Duct adenocarcinoma with necrosis	2	IB	T2N0M0	Malignant
PA961c	D8	F	46	Pancreas	Duct adenocarcinoma	2	IB	T2N0M0	Malignant
PA961c	D9	M	52	Pancreas	Duct adenocarcinoma	2	IB	T2N0M0	Malignant
PA961c	D10	M	52	Pancreas	Duct adenocarcinoma	2	IB	T2N0M0	Malignant
PA961c	D11	F	53	Pancreas	Duct adenocarcinoma	2	IIB	T2N1M0	Malignant
PA961c	D12	M	40	Pancreas	Duct adenocarcinoma	1	IIB	T3N1bM0	Malignant
PA961c	E1	M	57	Pancreas	Duct adenocarcinoma	1	IIA	T3N0M0	Malignant
PA961c	E2	M	31	Pancreas	Duct adenocarcinoma	1	IIA	T3N0M0	Malignant
PA961c	E3	M	44	Pancreas	Duct adenocarcinoma	2	IIA	T3N0M0	Malignant
PA961c	E4	M	61	Pancreas	Adenocarcinoma	3	IV	T3N0M1	Malignant
PA961c	E5	M	51	Pancreas	Duct adenocarcinoma	2	IIA	T3N0M0	Malignant
PA961c	E6	M	59	Pancreas	Duct adenocarcinoma	2	IA	T1N0M0	Malignant
PA961c	E7	M	44	Pancreas	Adenocarcinoma	3	IIA	T3N0M0	Malignant
PA961c	E8	M	45	Pancreas	Adenocarcinoma	3	IIB	T2N1M0	Malignant
PA961c	E9	M	41	Pancreas	Duct adenocarcinoma	2	IB	T2N0M0	Malignant
PA961c	E10	F	72	Pancreas	Duct adenocarcinoma	2	IIA	T3N0M0	Malignant
PA961c	E11	F	51	Pancreas	Duct adenocarcinoma	-	IIA	T3N0M0	Malignant
PA961c	E12	F	42	Pancreas	Duct adenocarcinoma	2	IB	T2N0M0	Malignant
PA961c	F1	F	39	Pancreas	Duct adenocarcinoma	2	IIA	T3N0M0	Malignant
PA961c	F2	F	51	Pancreas	Duct adenocarcinoma with necrosis	2	IIA	T3N0M0	Malignant
PA961c	F3	M	62	Pancreas	Duct adenocarcinoma	1	IIA	T3N0M0	Malignant
PA961c	F4	M	60	Pancreas	Duct adenocarcinoma	2	IB	T2N0M0	Malignant
PA961c	F5	F	53	Pancreas	Duct adenocarcinoma	2	IIA	T3N0M0	Malignant

PA961c	F6	M	77	Pancreas	Duct adenocarcinoma	2	IA	T1N0M0	Malignant
PA961c	F7	M	47	Pancreas	Adenocarcinoma	3	IIA	T3N0M0	Malignant
PA961c	F8	M	67	Pancreas	Adenocarcinoma	3	IIA	T3N0M0	Malignant
PA961c	F9	M	78	Pancreas	Adenocarcinoma	3	IB	T2N0M0	Malignant
PA961c	F10	M	62	Pancreas	Adenocarcinoma	3	IIA	T3N0M0	Malignant
PA961c	F11	M	50	Pancreas	Adenocarcinoma	3	IB	T2N0M0	Malignant
PA961c	F12	M	55	Pancreas	Adenocarcinoma	3	IIB	T3N1M0	Malignant
PA961c	G1	F	48	Pancreas	Adenocarcinoma	3	IIA	T3N0M0	Malignant
PA961c	G2	M	50	Pancreas	Adenocarcinoma	3	IIB	T3N1M0	Malignant
PA961c	G3	F	53	Pancreas	Adenocarcinoma with necrosis	3	IB	T2N0M0	Malignant
PA961c	G4	M	50	Pancreas	Adenocarcinoma	3	IB	T2N0M0	Malignant
PA961c	G5	F	23	Pancreas	Adenocarcinoma	3	IB	T2N0M0	Malignant
PA961c	G6	M	56	Pancreas	Adenocarcinoma	-	IIB	T2N1bM0	Malignant
PA961c	G7	F	56	Pancreas	Undifferentiated carcinoma	-	IIA	T3N0M0	Malignant
PA961c	G8	F	54	Pancreas	Mixed acinar-neuroendocrine carcinoma	-	IB	T2N0M0	Malignant
PA961c	G9	M	52	Pancreas	Adenosquamous carcinoma	-	IIA	T3N0M0	Malignant
PA961c	G10	F	49	Pancreas	Adenosquamous carcinoma	-	IIB	T3N1M0	Malignant
PA961c	G11	M	50	Pancreas	Adenosquamous carcinoma	-	IIA	T3N0M0	Malignant
PA961c	G12	M	62	Pancreas	Squamous cell carcinoma	2	IIA	T3N0M0	Malignant
PA961c	H1	F	52	Pancreas	Carcinoid	-	IIA	T3N0M0	Malignant
PA961c	H2	F	50	Pancreas	Carcinoid	-	IIA	T3N0M0	Malignant
PA961c	H3	M	51	Pancreas	Atypical carcinoid	-	III	T4N0M0	Malignant
PA961c	H4	M	42	Pancreas	Neuroendocrine carcinoma	-	IIA	T3N0M0	Malignant
PA961c	H5	M	53	Pancreas	Acinic cell carcinoma	-	IB	T2N0M0	Malignant
PA961c	H6	F	33	Pancreas	Solid pseudo-papillary carcinoma (chronic inflammation of pancreas tissue)	-	IIA	T3N0M0	Malignant
PA961c	H7	M	42	Pancreas	Solid pseudo-papillary carcinoma	-	IB	T2N0M0	Malignant
PA961c	H8	F	21	Pancreas	Pancreatic tissue	-	-	-	Normal
PA961c	H9	M	47	Pancreas	Pancreatic tissue	-	-	-	Normal
PA961c	H10	M	38	Pancreas	Pancreatic tissue	-	-	-	Normal
PA961c	H11	M	40	Pancreas	Pancreatic tissue	-	-	-	Normal
PA961c	H12	F	38	Pancreas	Pancreatic tissue	-	-	-	Normal

Appendix References

Pinho AV, Rooman I, Reichert M, De Medts N, Bouwens L, Rustgi AK, Real FX (2011) Adult pancreatic acinar cells dedifferentiate to an embryonic progenitor phenotype with concomitant activation of a senescence programme that is present in chronic pancreatitis. *Gut* **60:** 958-966
