# Supplementary material for: ROCK signaling promotes collagen remodeling to facilitate invasive pancreatic ductal adenocarcinoma tumor cell growth
Source: EMBO Mol Med. 2016 Dec 28;9(2):198–218. doi: 10.15252/emmm.201606743 (PMC5286371; doi:10.15252/emmm.201606743)
Supplement: Supplementary file 2 — Expanded View Figures PDF [file EMMM-9-198-s002.pdf]

Expanded View Figures

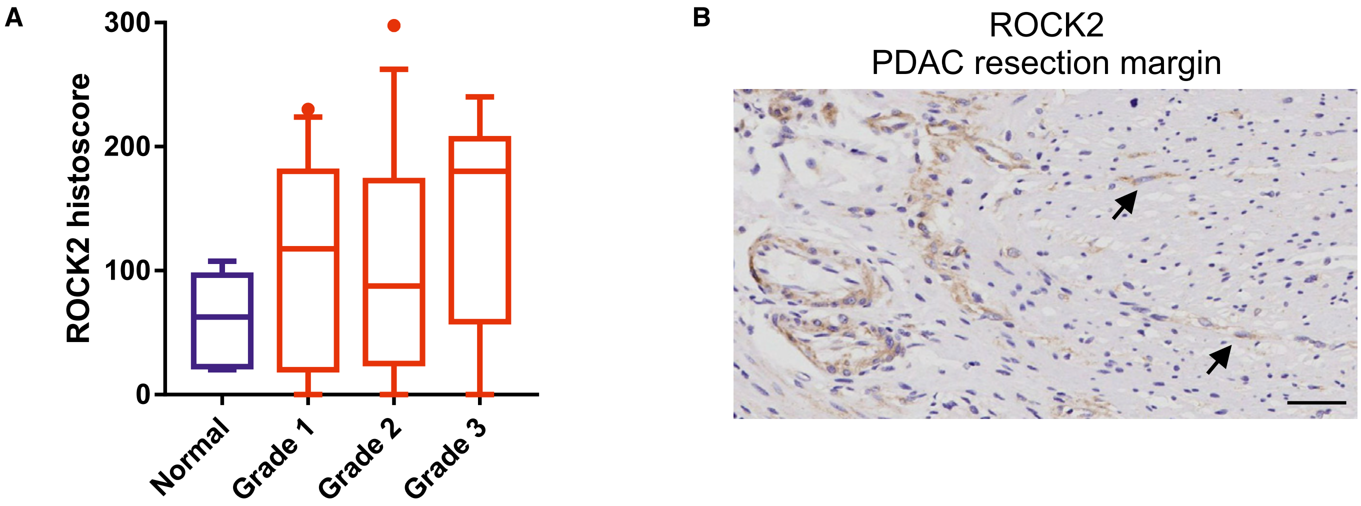

Figure EV1. ROCK2 association with pancreas adenocarcinoma grade and expression in tumor cells in resection margin (related to Fig 1).

A Histoscores of ROCK2 staining (Fig 1A) in normal pancreas (n = 5), and pancreas adenocarcinoma grade 1 (n = 23), grade 2 (n = 27), and grade 3 (n = 13). Box (upper and lower quartiles divided by median value) and whisker (5th–95th percentile) plots show outliers as individual points.

B ROCK2-positive stained cells (indicated with arrows) found beyond the tumor in the resection margin of human PDAC.

 © 2016 Cancer Research UK Beatson Institute

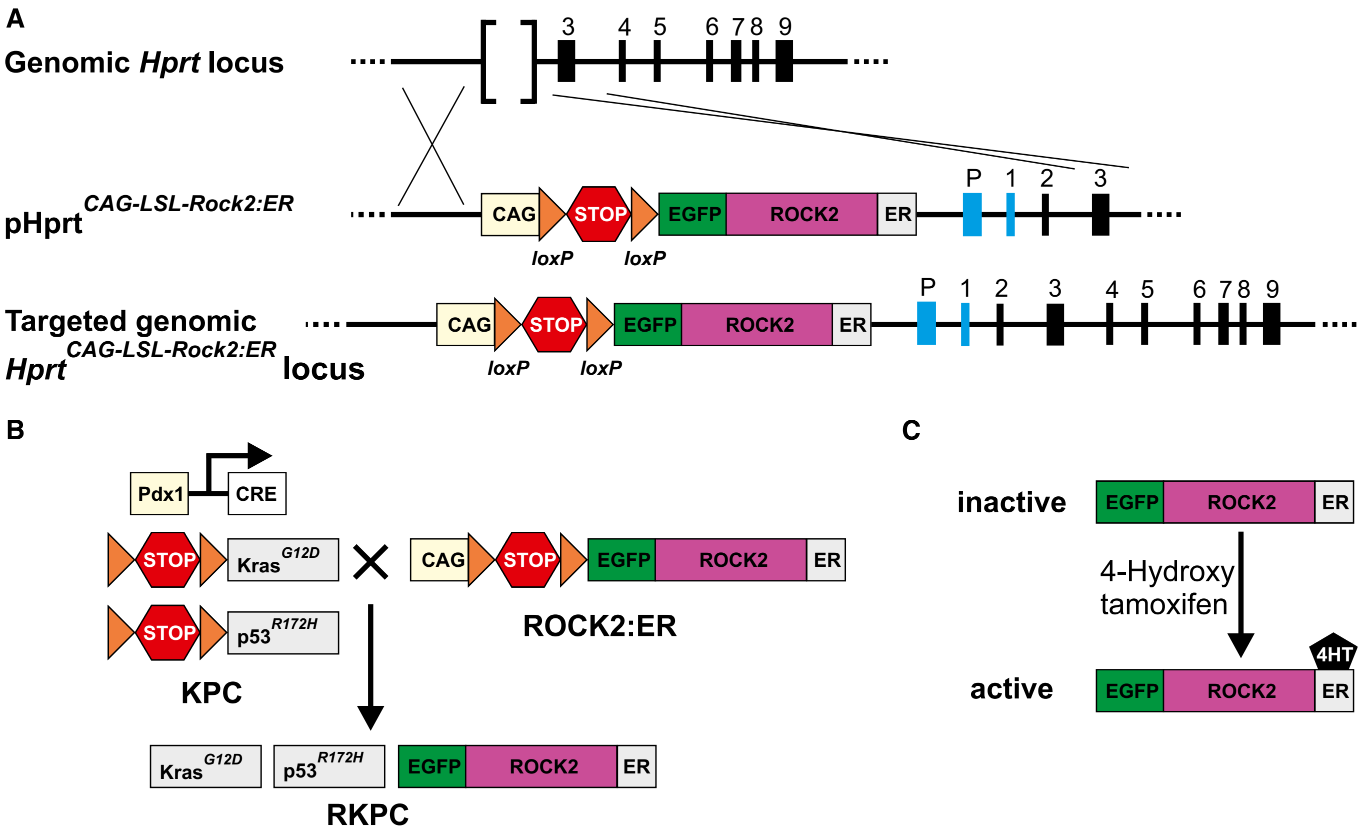

Figure EV2. Tissue-selective expression of conditionally activated ROCK2 (related to Fig 1).

A A *CAG-LSL-Rock2:ER* expression cassette was targeted to the mutant mouse *Hprt* gene locus together with the promoter and first exon of human *HPRT* and the second exon of murine *Hprt* to reconstitute an active chimeric *Hprt* locus. Not to scale.

B Generation of LSL-ROCK2:ER; KPC (RKPC) mice.

C Conditional activation of the ROCK2:ER fusion protein.

Figure EV3. ROCK-induced collagen matrix invasion (related to Fig 2).

A H&E-stained sections of cell invasion into collagen matrix containing embedded fibroblasts after 8 days. Scale bar = 100 µm. Invasion index of KPflC cells treated with 1 µM 4HT. Means ± SEM (n = 6), one-way ANOVA with multiplicity adjusted exact P-value by *post hoc* Dunnett's multiple comparison test.

B H&E-stained sections of cell invasion into collagen matrix after 8 days. Scale bar = 100 µm. Invasion index of KPflC cells treated with EtOH vehicle, 1 µM 4HT, or 1 µM 4HT + 10 µM H1152. Means ± SEM (n = 6; n = 5 for ROCK2:ER/4HT), one-way ANOVA with multiplicity adjusted exact P-value by *post hoc* Tukey multiple comparison test.

C Cell proliferation determined by Ki67 immunofluorescence. Scale bar = 20 µm.

D Quantification of cell number at the collagen matrix surface per 0.046 mm^2 field. Means ± SEM (n = 24), one-way ANOVA with multiplicity adjusted exact P-value by *post hoc* Tukey multiple comparison test.

E Ki67-positive cell percentages at the surface. Means ± SEM (n = 24), one-way ANOVA with multiplicity adjusted exact P-value by *post hoc* Tukey multiple comparison test.

© 2016 Cancer Research UK Beatson Institute

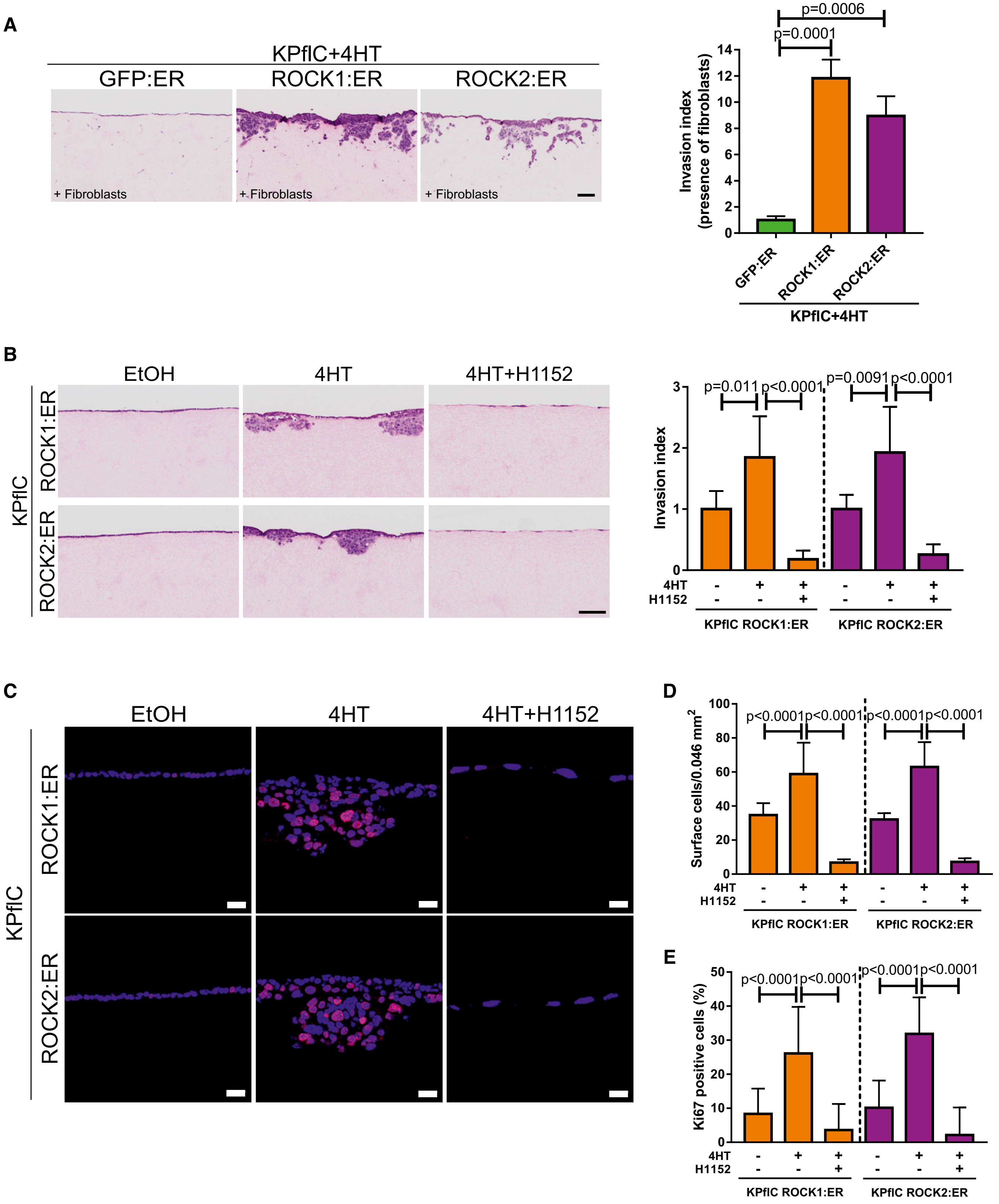

Figure EV3.

 © 2016 Cancer Research UK Beatson Institute

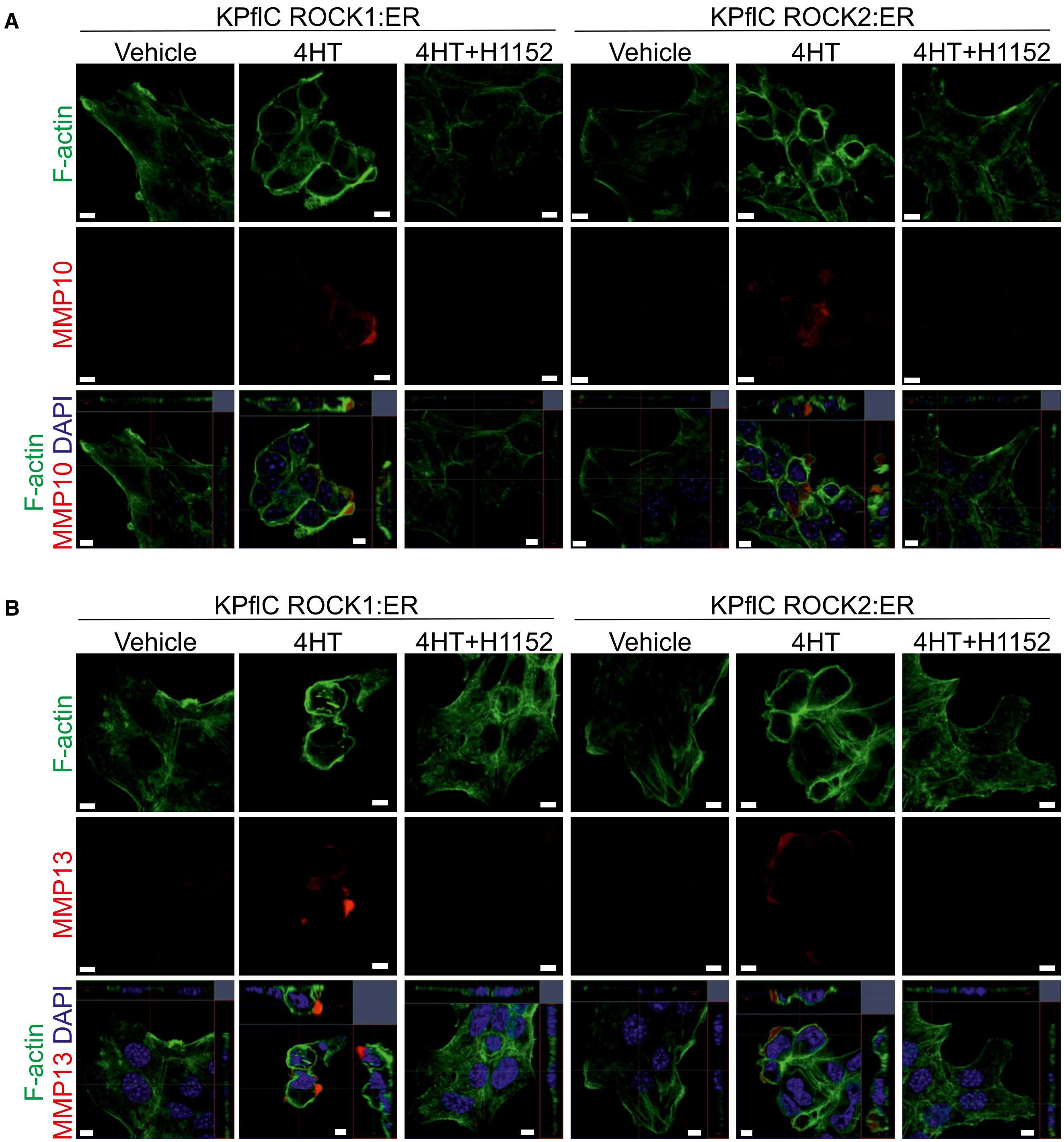

Figure EV4. Accumulation of MMP10 and MMP13 in bleblike protrusions (related to Fig 5).

A, B Confocal microscope images of KPflC cells expressing ROCK1:ER or ROCK2:ER as indicated co-stained for F-actin, MMP10 (A) or MMP13 (B), and DAPI following treatment with vehicle, 1 μM 4HT, or 1 μM 4HT + 1 μM H1152 for 24 h. Multiple *z*-planes were used to generate *x*–*z* and *y*–*z* images (bottom). Scale bar = 5 μm.

© 2016 Cancer Research UK Beatson Institute
