# Supplementary figures and images for: ROCK signaling promotes collagen remodeling to facilitate invasive pancreatic ductal adenocarcinoma tumor cell growth
Source: EMBO Mol Med. 2016 Dec 28;9(2):198–218. doi: 10.15252/emmm.201606743 (PMC5286371; doi:10.15252/emmm.201606743)

Source Data for Figure 2

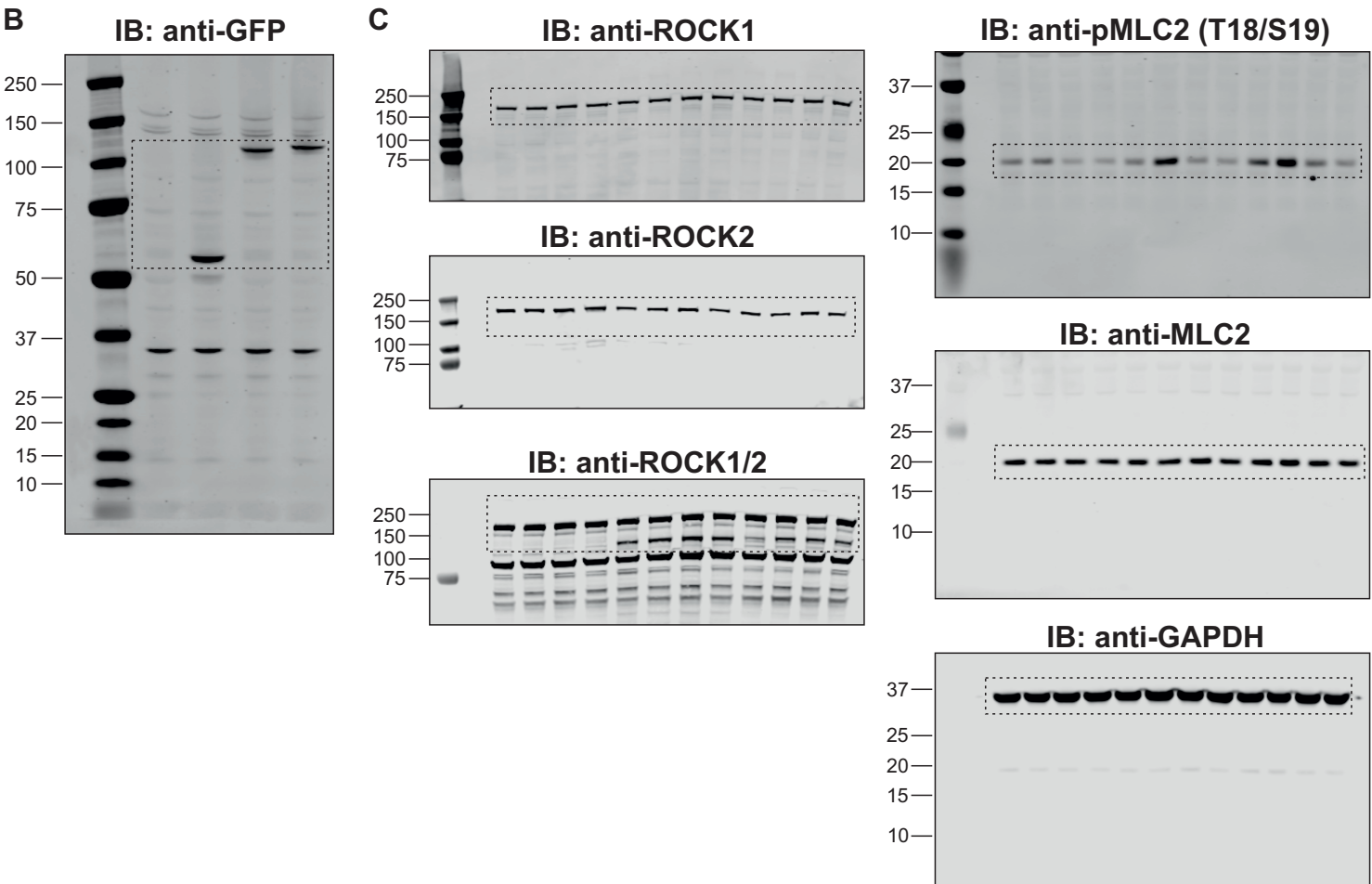

Supplement: Supplementary file 5 — Source Data for Figure 2 [file EMMM-9-198-s004.pdf]

Source Data for Figure 5

B

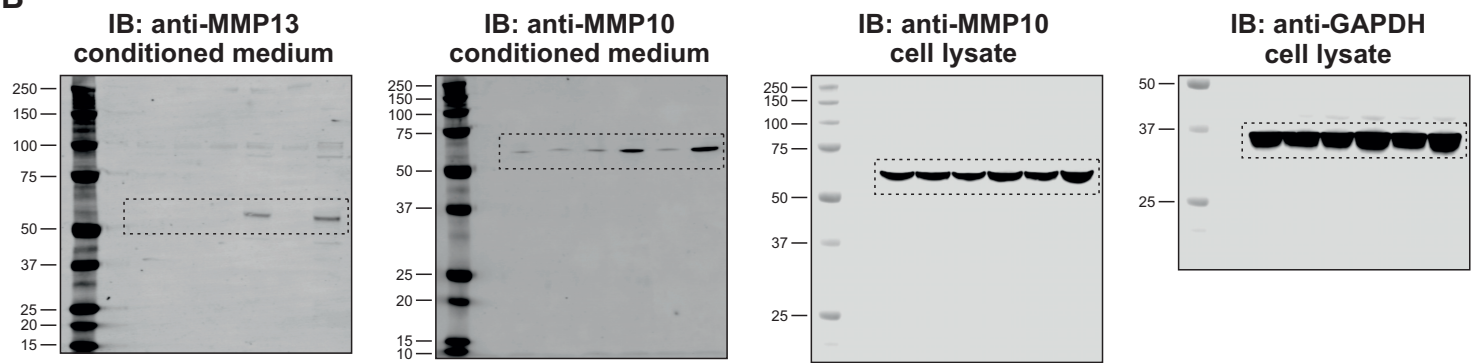

D

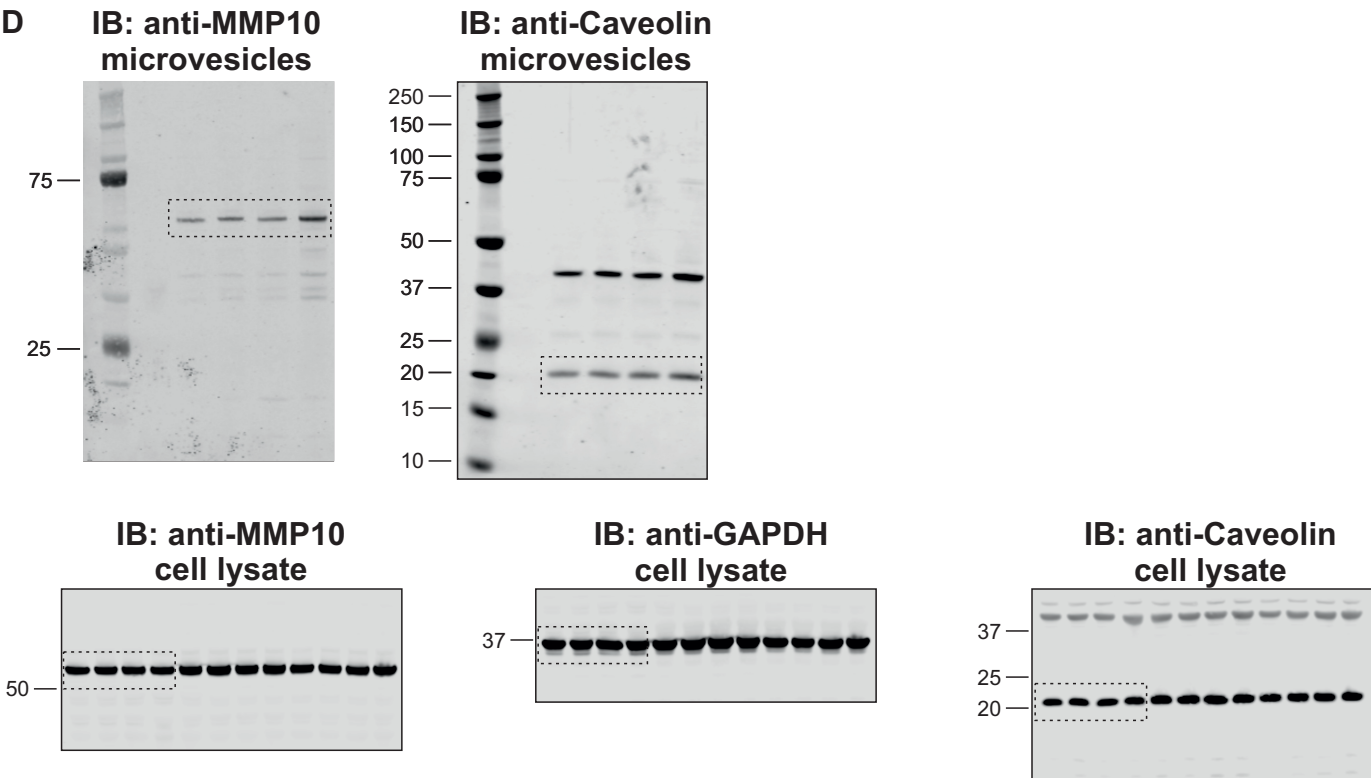

Supplement: Supplementary file 6 — Source Data for Figure 5 [file EMMM-9-198-s005.pdf]
